# Enhancing collective entanglement witnesses through correlation with state purity

**DOI:** 10.1038/s41598-024-65385-7

**Published:** 2024-07-16

**Authors:** Kateřina Jiráková, Antonín Černoch, Artur Barasiński, Karel Lemr

**Affiliations:** 1 Institute of Physics of the Academy of Sciences of the Czech Republic, Joint Laboratory of Optics of Palacký University and Institute of Physics AS CR, 17. listopadu 50a, 772 07 Olomouc, Czech Republic; 2 Institute of Theoretical Physics, University of Wroclaw, Plac Maxa Borna 9, 50-204 Wroclaw, Poland; 3 Joint Laboratory of Optics of Palacký University, Institute of Physics AS CR, Faculty of Science, Palacký University in Olomouc, 17. listopadu 12, 771 46 Olomouc, Czech Republic

**Keywords:** Quantum physics, Quantum information, Qubits

## Abstract

This research analyzes the adverse impact of white noise on collective quantum measurements and argues that such noise poses a significant obstacle for the otherwise straightforward deployment of collective measurements in quantum communications. Our findings then suggests addressing this issue by correlating outcomes of these measurements with quantum state purity. To test the concept, a support vector machine is employed to boost the performance of several collective entanglement witnesses by incorporating state purity into the classification task of distinguishing entangled states from separable ones. Furthermore, the application of machine learning allows to optimize specificity of entanglement detection given a target value of sensitivity. A response operating characteristic curve is reconstructed based on this optimization and the area under curve calculated to assess the efficacy of the proposed model. Finally, we test the presented approach on an experimental dataset of Werner states.

## Introduction

**Figure 1:**
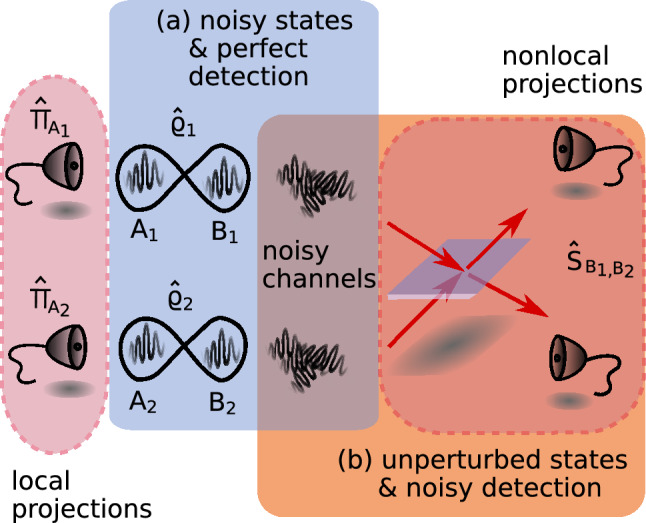
Conceptual scheme of collective measurement with $n = 1$ nonlocal projections. Imperfections in the setup can be modelled by insertion of two noisy channels. The effect of these channels can be described by two equivalent strategies: (**a**) the two states $\hat{\rho }_1$ and $\hat{\rho }_2$ become noisy while the nonlocal measurement remains perfect or (**b**) perfect unperturbed states are subject to imperfect nonlocal projection.

Collective quantum measurements, which are measurements performed simultaneously on multiple copies of the investigated state, are an invaluable tool in quantum state analysis^1^. Since the pioneering experimental demonstration in 2005^2^, collective measurements have been instrumental in practical implementation of a number of entanglement witnesses^3–11^ and to infer quantum state purity or fidelity^12,13^. The key advantage of these measurements lies in the projection of subsystems from different copies of the examined state onto a common entangled state (see nonlocal projection in conceptual diagram in Fig. 1). Thanks to these nonlocal projections, collective measurements outperform ordinary single-copy measurements in terms of sensitivity (e.g. the volume of detected entangled states^5^) or in the efficiency in achieving set tasks with fewer measurements^9,10^. Notably, the required number of collective measurements does not grow as prohibitively fast with the size of the Hilbert space as is the case with standard quantum state tomography.

Application-wise, collective measurements hold particular appeal in conjunction with entanglement distribution in quantum networks. Their layout (visualized in Fig. 1) aligns with the topology of entanglement swapping making collective measurements straightforwardly deployable in quantum repeaters^14^, relays^15^ and in teleportation-based quantum communications networks^16,17^. For instance, research has demonstrated applicability of collective measurements for a rapid and practical diagnostics of the entanglement swapping protocol^18^. Moreover, direct measurement of Hilbert-Schmidt distance through collective measurements has been used to speed-up K-means classification algorithm^12^. Usage of these collective measurements may also prove interesting beyond optical platforms, considering that light can facilitate entanglement detection in other physical system as well^19–21^.

As explained above, the power of collective measurements lies in the nonlocal projections. However, these projections are notably susceptible to white noise, and consequently, utilization of collective measurements in real-world quantum communications necessitates an assessment of their performance in the presence of that noise. In section Methods of this paper, we analyze propagation of the investigated state through noisy channel and document the adverse effect of the channel on collective measurements as the noise accumulates polynomially fast with the power corresponding to the number of copies projected onto a shared entangled state. Hence even a rather weakly perturbing realistic quantum channel can cause the projections to become local (no longer projecting on an entangled state) and, thus, the entire collective measurement looses its power. This effect is exemplified on the incapability of collectibility (a collective entanglement witness) to correctly detect entangled Werner state with a noise level above $p = 1-\tfrac{\sqrt{3}}{2} \approx 0.13$^4^.

Simultaneously, white noise is commonly associated with a reduction in the purity of transmitted quantum states. In this paper, we analyze the correlation between collective entanglement witnesses and purity of the states aiming to mitigate the influence of white noise on collective measurements. Our findings reveal that this way one can considerably enhance sensitivity of collective entanglement witnesses (true positive rate^22,23^ or TPR) at a relatively small cost in decreased specificity (increased false positive rate or FPR). A support vector machine is employed to find the optimal classification boundary between entangled and separable states, maximizing specificity for a given value of sensitivity boost (see Section States generation and classification). Furthermore, targeting several sensitivity values and identifying the corresponding specificity allows us to reconstruct the entire receiver operating characteristic (ROC) curve, evaluating the method’s effectiveness in terms of the area under this curve (AUC). Note that reconstructing the ROC curve would not be possible without the use of machine learning as analytical entanglement witnesses assume perfect specificity (FPR$=0$). However, such assumption is unrealistic due to the inevitable technical imperfections in practical conditions, at least such as detection shot noise^24^. Our analysis of the ROCs contributes to the assessment of practicality of collective entanglement witnesses for near-future quantum communications networks.

## Vulnerability of collective measurements to white noise

In this section, we demonstrate that collective measurements are vulnerable to the presence of noise, in particular white noise. The sensitivity of entanglement swapping towards several kinds of noise has already been studied thoroughly in the literature^25–28^. On the other hand, we intend to show particularly the impact of the white noise on the collective entanglement witnesses by deriving explicit formulas. Let us consider a simple scenario illustrated in the Fig. 1 and assume that two copies of a Bell state $\hat{\rho }_{1} = \hat{\rho }_{2} = \hat{\text {P}}^+=|\phi ^{+} \rangle \langle \phi ^{+}|$ ($|\phi ^{+}\rangle =\frac{1}{\sqrt{2}} \sum _{i=0}^1 |ii\rangle$) are subject to the collective measurement.

Imperfections in the procedure are modelled by two channels introducing the noise with probability *p*. There are two equivalent strategies that can be used to incorporate the effect of noise on the outcome of collective measurements. In strategy (a) we add noise to the investigated states $\hat{\rho }_{1}$, $\hat{\rho }_{2}$ that are then tested in a perfect collective measurement. In the second strategy (b), both noisy channels are added to the nonlocal measurement projector $\hat{S}_{\text {B}_{\text {1}}, \text {B}_{\text {2}}}$ turning it into a POVM. In this strategy, perfect Bell states $\hat{\rho }_{i}$ are measured by means of this POVM.

To prove this, let us first define the probabilities of collective projections, which in the next section will be used as a central quantity in the analyzed nonclassical witnesses. The probabilities of collective projections are given by

$$\begin{aligned} C (\hat{\Pi }_{\text {A}_{\text {1}}},\hat{\Pi }_{\text {A}_{\text {2}}}) = \text {Tr}\left[ \left( \hat{\chi }_{\text {A}_{\text {1}}, \text {B}_{\text {1}}} \otimes \hat{\chi }_{\text {A}_{\text {2}}, \text {B}_{\text {2}}}\right) \left( \hat{S}_{\text {B}_{\text {1}}, \text {B}_{\text {2}}}\otimes \hat{\Pi }_{\text {A}_{\text {1}}}\otimes \hat{\Pi }_{\text {A}_{\text {2}}}\right) \right] \end{aligned}$$

and

$$\begin{aligned} \bar{C} (\hat{\Pi }_{\text {A}_{\text {1}}},\hat{\Pi }_{\text {A}_{\text {2}}}) = \text {Tr}\left[ \left( \hat{\chi }_{\text {A}_{\text {1}}, \text {B}_{\text {1}}} \otimes \hat{\chi }_{\text {A}_{\text {2}}, \text {B}_{\text {2}}}\right) \left( \hat{\mathbbm {1}}_4 \otimes \hat{\Pi }_{\text {A}_{\text {1}}}\otimes \hat{\Pi }_{\text {A}_{\text {2}}}\right) \right] , \end{aligned}$$

where $\hat{\Pi }_{i}$ stands for local projection, $\hat{S}_{\text {B}_{\text {1}}, \text {B}_{\text {2}}}$ represents the two-qubit collective measurement, and $\hat{\chi }_{\text {A}_{\text {i}}, \text {B}_{\text {i}}}$ denotes the Bell state $\hat{\rho }_{i}$ already affected by the noise. Here, indexes $\text {A}_{i}$ and $\text {B}_{i}$ are used to denote the first and the second qubit of analyzed state.

Note that by virtue of the Jamiołkowski isomorphism^29^, the noisy channel $\hat{\chi }$ can be written as $(\hat{\mathbbm {1}} \otimes \varvec{\Gamma })[\hat{\text {P}}^+]$, with $\varvec{\Gamma }[\cdot ]:=\sum _k \Gamma ^{(k)} \cdot (\Gamma ^{(k)})^{\dagger }$ a completely positive map (see e.g.^30^). Then, with straightforward calculations, one can write

1 $$\begin{aligned} \hat{\chi }_{\text {A}_{\text {1}}, \text {B}_{\text {1}}} \otimes \hat{\chi }_{\text {A}_{\text {2}}, \text {B}_{\text {2}}} = \sum _{k_1,k_2} \left( \hat{\mathbbm {1}}_{\text {A}_{\text {1}}}\otimes \Gamma ^{(k_1)}_{\text {B}_{\text {1}}}\otimes \hat{\mathbbm {1}}_{\text {A}_{\text {2}}}\otimes \Gamma ^{(k_2)}_{\text {B}_{\text {2}}}\right) \times \left( \hat{\text {P}}^+_{\text {A}_{\text {1}}, \text {B}_{\text {1}}} \otimes \hat{\text {P}}^+_{\text {A}_{\text {2}}, \text {B}_{\text {2}}}\right) \left( \hat{\mathbbm {1}}_{\text {A}_{\text {1}}}\otimes \Gamma ^{(k_1)}_{\text {B}_{\text {1}}}\otimes \hat{\mathbbm {1}}_{\text {A}_{\text {2}}}\otimes \Gamma ^{(k_2)}_{\text {B}_{\text {2}}}\right) ^{\dagger }. \end{aligned}$$

Using this equation and the cyclic property of the trace function, one gets

$$\begin{aligned} C (\hat{\Pi }_{\text {A}_{\text {1}}},\hat{\Pi }_{\text {A}_{\text {2}}}) = \text {Tr}\left[ \left( \hat{\text {P}}^+_{\text {A}_{\text {1}}, \text {B}_{\text {1}}} \otimes \hat{\text {P}}^+_{\text {A}_{\text {2}}, \text {B}_{\text {2}}}\right) \left( \hat{\Omega }_{\text {B}_{\text {1}}, \text {B}_{\text {2}}}\otimes \hat{\Pi }_{\text {A}_{\text {1}}}\otimes \hat{\Pi }_{\text {A}_{\text {2}}}\right) \right] , \end{aligned}$$

where the noisy collective measurement $\hat{\Omega }_{\text {B}_{\text {1}}, \text {B}_{\text {2}}}= \sum _{k_1,k_2} \left( \Gamma ^{(k_1)}_{\text {B}_{\text {1}}}\otimes \Gamma ^{(k_2)}_{\text {B}_{\text {2}}}\right) ^{\dagger } \hat{S}_{\text {B}_{\text {1}}, \text {B}_{\text {2}}} \left( \Gamma ^{(k_1)}_{\text {B}_{\text {1}}}\otimes \Gamma ^{(k_2)}_{\text {B}_{\text {2}}}\right)$ is the mathematical representation of the strategy (b). Note that $\hat{\Omega }_{\text {B}_{\text {1}}, \text {B}_{\text {2}}}$ accumulates noise from both channels. Similar calculations can be made for $\bar{C} (\hat{\Pi }_{\text {A}_{\text {1}}},\hat{\Pi }_{\text {A}_{\text {2}}})$ showing that this quantity is, however, invariant to noise as $\varvec{\Gamma }$ is trace-preserving (i.e. $\sum _k (\Gamma ^{(k)})^{\dagger } \Gamma ^{(k)}=\hat{\mathbbm {1}}$). Therefore, scenario (a) is equivalent to scenario (b) when $\{\hat{\chi }_{\text {A}_{\text {1}}, \text {B}_{\text {1}}}, \hat{\chi }_{\text {A}_{\text {2}}, \text {B}_{\text {2}}},\hat{S}_{\text {B}_{\text {1}}, \text {B}_{\text {2}}}\} \leftrightarrow \{\hat{\text {P}}^+_{\text {A}_{\text {1}}, \text {B}_{\text {1}}}, \hat{\text {P}}^+_{\text {A}_{\text {2}}, \text {B}_{\text {2}}},\hat{\Omega }_{\text {B}_{\text {1}}, \text {B}_{\text {2}}}\}$. It is worth mentioning that the above considerations apply to an arbitrary state $\chi$, not just noisy Bell states.

Let us now consider a particular, yet practical, situation when $\varvec{\Gamma }$ stands for a depolarizing channel^31^ and $\hat{S}_{\text {B}_{\text {1}}, \text {B}_{\text {2}}} = \hat{\text {P}}^+_{\text {B}_{\text {1}}, \text {B}_{\text {2}}}$. In this case, the Bell state is transformed into the Werner state with visibility $v=(1-p)$

2 $$\begin{aligned} \hat{\text {P}}^+ \longrightarrow \chi =\hat{\rho }_{\text {W}}(p) = (1-p)~\hat{\text {P}}^+ + p\frac{\hat{\mathbbm {1}}}{4}. \end{aligned}$$

On the other hand, in the second strategy (b) one gets

3 $$\begin{aligned} \hat{S}_{\text {B}_{\text {1}}, \text {B}_{\text {2}}} = \hat{\text {P}}^+ \longrightarrow \hat{\Omega }_{\text {B}_{\text {1}}, \text {B}_{\text {2}}} = (1-p)^{2}~\hat{\text {P}}^+ + (2-p)p\frac{\hat{\mathbbm {1}}}{4} \equiv \hat{\rho }_{\text {W}}\Big ((1-p)^2\Big ), \end{aligned}$$

i.e. a POVM described by the Werner state with visibility $v = (1-p)^{2}$.

Consider that the Werner state becomes separable for $v \le \tfrac{1}{3}$ and that projecting on a separable state removes the advantage of collective measurements as such measurement can be decoupled into a series of local single-state copy measurements. The threshold $v = \tfrac{1}{3}$ is reached if the channel introduces noise with probability $p = 1-\sqrt{\tfrac{1}{3}} \approx 0.42$. As a consequence, entanglement witnesses based on collective measurement thus rather quickly lose their detection power when white noise is introduced. Note that for instance one of these witnesses, the collectibility, does not even detect all Bell nonlocal Werner states given by $v_C > \tfrac{1}{\sqrt{2}} \approx 0.71$^32^. Collectibility only detects nonlocal correlations for Werner states with $v_W > \tfrac{\sqrt{3}}{2} \approx 0.87$^4^ while the entropic witness is limited by $v_E >\frac{1}{\sqrt{3}}$.

## Methods

As demonstrated in the previous section, white noise has a negative effect on sensitivity of collective entanglement witnesses. Simultaneously, white noise also causes purity of examined quantum states to decrease. This leads us to propose the idea of restoring the performance of collective witnesses by correlating their values with purity of the examined states. To test this idea, the following three steps were implemented: (i) generation of random two-qubit states with uniform distribution in purity, (ii) estimation of three collective entanglement witnesses and purity of the state, (iii) classification of the states via machine learning.

### States generation

First, we have generated random two-qubit states following the method described in Ref.^9,33,34^ and also explained in detail in the Supplementary Material. This method is based on the preparation of a diagonal matrix with uniformly distributed eigenvalues which is then subjected to a random two-qubit unitary evolution. The appropriate SU(4) unitary matrices are generated according to a procedure described in Ref.^35^. Although such approach provides a uniform distribution of states with respect to the Haar measure, it does not provide a uniform distribution of state purity. There are also other methods for random state generation proposed in literature each leading to a different sampling of the states hence a different distribution of their purities^36,37^. To asses the distribution of sampled states using various algorithms, we have calculated histograms of Hilbert-Schmidt distances among all combinations of 10 000 random states sampled using four different algorithms^36,37^. The results of our analysis are depicted in Supplementary Fig. 1 of the Supplementary Material allowing to identify that the selected method based on generation of a random diagonal matrix subjected afterwards to a random global rotation^9,33^ leads to the highest spread of the Hilbert-Schmidt distance values. This indicates that this method provides states with the best coverage of the Hilbert space minimizing thus a potential bias of any machine learning on these states.

To even further tackle the issue of a relatively small contribution of quasi-pure states in the samples, we have decided to post-select from the generated states a subset of 2 million states with uniform distribution of purity. We consider this subset useful for benchmarking as it has considerably increased the presence of pure and quasi-pure states with respect to all tested vanilla random state generation algorithms. As a result, it favours entanglement detection based solely on the entanglement witnesses *without* correlating them with purity and hence the improvement obtained by incorporating purity into the decision is rather underestimated and certainly not overestimated.

**Figure 2:**
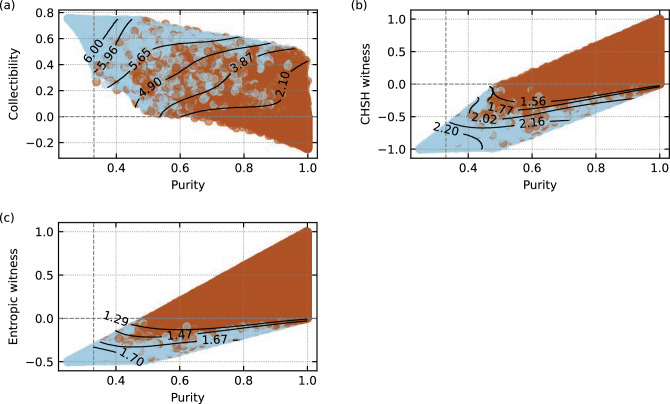
Distribution of separable (light blue) and entangled (dark reddish) states in the feature space of the collective witnesses and purity is depicted for the three evaluated witnesses: (**a**) collectibility, (**b**) the CHSH witness, and (**c**) the entropic witness. As a sanity check, the analytical decision threshold at 0 is marked by the horizontal dashed line. Similarly the purity threshold below which all states must be separable at 1/3^4^ is marked by vertical dashed line. Selected support vector machine decision boundaries are plotted by solid black lines labelled by the corresponding improvement factors IF. Note that for clarity of these plots, not all contours (each corresponding to a particular selection of the penalties pair $w_e$ and $w_s$) are plotted. Supplementary Tables 2–4 in the Supplementary Material contain numerical data for all penalties.

### Entanglement witnesses and purity

With the random states generated, we have calculated values of three typical collective entanglement witnesses that can be measured in the entanglement swapping geometry: (a) the collectibility, (b) the CHSH witness, and (c) the entropic witness. The Collectibility as used in this article is defined as

4 $$\begin{aligned} W = \frac{1}{2}\left[ \eta + X^2_0(1-2 X_{00}) + X^2_1(1-2 X_{11}) + 2 X_0X_1(1-2 X_{01}) -1 \right] \end{aligned}$$

where

5 $$\begin{aligned} \eta = 16 X_{0} X_{1}\sqrt{X_{00} X_{11}} + 4 \cdot \text {max} \{X_{++}, X_{--}\} \end{aligned}$$

and

6 $$\begin{aligned} X_{ij} = \frac{ C(\hat{\Pi }_{{i}},\hat{\Pi }_{{j}})}{\bar{C} (\hat{\Pi }_{{i}},\hat{\Pi }_{{j}})}, \hspace{2mm} X_0 = 1 - X_1 = \bar{C} (\hat{\Pi }_{\text {0}},\hat{\Pi }_{\text {0}}) + \bar{C} (\hat{\Pi }_{\text {0}},\hat{\Pi }_{\text {1}}), \end{aligned}$$

with $i, j \in \{0, 1, +, -\}$ denote logical and Hadamard basis states^4^. Moreover, with the correlation matrix defined as^38^

7 $$\begin{aligned} R_{{m,n}} = \bar{C}(\sigma _{{m}}, \sigma _{{n}}) - 4 C(\sigma _{{m}}, \sigma _{{n}}), \end{aligned}$$

where $\sigma _{{m}}$ and $\sigma _{{n}}$ are Pauli operators, one can calculate the entropic witness^2,6^ as

8 $$\begin{aligned} \text{EW}(\hat{\rho }) = \frac{1}{2}\Big [\text {Tr}(R) - 1 \Big ] \end{aligned}$$

and also the CHSH witness in the form^39,40^

9 $$\begin{aligned} \text{CHSH}(\hat{\rho }) = \text {Tr}(R) - \text {min[eig }(R)] - 1. \end{aligned}$$

Purity of each state was also calculated. Note that purity can be directly measured using collective measurement on two copies of the investigated state using either the approach involving $n = 2$ nonlocal projections^2,41^

$$\begin{aligned} P (\hat{\rho }) = \text {Tr}\left[ \hat{\rho }_{\text {A}_{\text {1}}, \text {B}_{\text {1}}} \otimes \hat{\rho }_{\text {A}_{\text {2}}, \text {B}_{\text {2}}} (1-2S)_{\text {A}_{\text {1}}, \text {A}_{\text {2}}} \otimes (1-2S)_{\text {B}_{\text {1}}, \text {B}_{\text {2}}}\right] \end{aligned}$$

or in the entanglement swapping geometry following the idea to decompose nonlocal projection into a series of local measurements

10 $$\begin{aligned} \hat{S}_{\text {A}_{\text {1}}, \text {A}_{\text {2}}} = \frac{1}{4}\Big (1- \sum _{{m}} \sigma _{{m}} \otimes \sigma _{{m}}\Big )_{\text {A}_{\text {1}}, \text {A}_{\text {2}}} \end{aligned}$$

described in Ref.^38^.

### Classification of states

In the third step, each collective witness is paired with purity to obtain sets of feature vectors of length 2 to be then used for classification via machine learning (one set per witness). True labels for training were provided by the PPT criterion which is, for two-qubit states, a sufficient and necessary condition for entanglement. Note that in practical conditions the PPT criterion requires complete quantum state tomography to be performed and hence also the number of measurements quadratically growing with the size of the Hilbert space^42^. Furthermore, negativity cannot be measured in a collective measurement on two copies of the investigated state^5^. Distribution of the feature vectors for all three witnesses colored based on their true label is visualized in Fig. 2a–c. Classification decision based solely on the analytical formulae of the three witnesses is visualized in these figures by dashed horizontal lines. Similarly, the states with purities $< 1/3$ that cannot be entangled are delimited by a vertical dashed line^4^.

Incorporating purity into the classification of the states and simultaneously tuning the required sensitivity and specificity is a task that can not be tackled by means of analytical calculations. We thus resort to a machine learning-based approach. For all three witnesses, the set of feature vectors was split into two halves, one used to train support vector machine classifiers (SVC) and the other to test their performance. After experimenting with the hyper-parameters, we have decided to use the radial basis function (RBF) kernels with $\gamma =1$. To speed-up the training, final classification of the test set is based on 11 hard-voting SVCs each trained on 1/11 of the training instances. To observe complete ROC curves, SVC were trained with 12 different class-based penalties^43^ for misclassification of entangled states $w_\text{e}$ ranging from $10^{0.75}$ to $10^{-1.15}$. Penalty for misclassification of separable states $w_\text{s}$ was adjusted always so that $w_\text{s} w_\text{e} = 1$ to maintain regularization constant. Specific values of these penalties are presented in the Supplementary Material. Their choice affects the sensitivity and specificity of the SVC. We have tuned their values so that the entire ROC curves are homogeneously covered allowing also for a good estimate of the areas under curves. It should be emphasized that the penalties are parameters that users of our method can freely tune (and subsequently retrain the models) to suit their particular needs. To benchmark sensitivity, we have calculated the APR (analytical positive rate), i.e. the percentage of entangled states that a given witness is capable of identifying solely based on the analytical threshold without the usage of purity. Sensitivity of each SVC is then characterized by its TPR (true positive rate) and the resulting improvement factor (IF)

11 $${\text{IF}} = \frac{{\text{TPR}}}{{\text{APR}}}.$$

Specificity, on the other hand, is directly described by the false positive rate (FPR). Note that decisions based solely on the analytical formulae of the witnesses (without the SVC) have $\text{FPR}=0$. Complete programming code in Python 3 using the ThunderSVM library^44^ is available as Digital Supplement^45^.

## Results

### Classification of general two-qubit states and the resulting ROC curves

For all combinations of the three witnesses and the 12 values of the class-based penalties $w_\text{e}$, the SVC learned an optimal boundary between separable and entangled states in the feature space. Selection of these boundaries is depicted in Fig. 2a–c together with the corresponding improvement factors IF.

**Figure 3:**
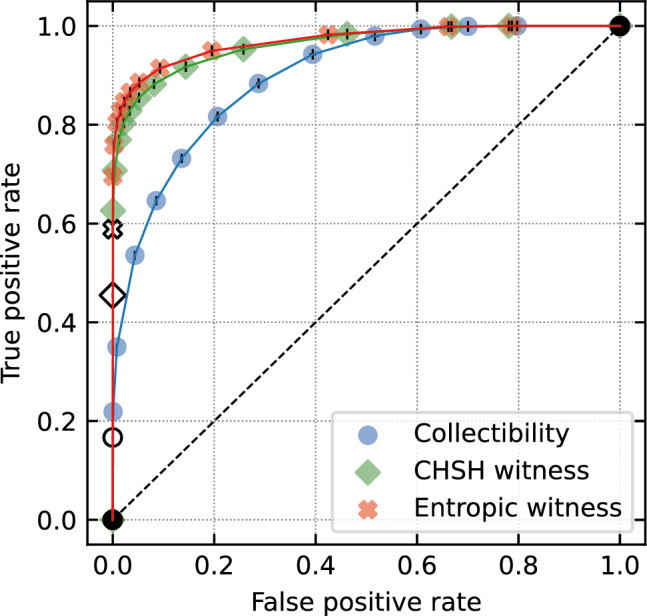
ROC curves for the purity-enhanced classification based on the three tested witnessed. Diagonal dashed line represents a naive (random) decision making while the filled markers correspond to SVCs trained with varying class-based penalties $w_\text{e}$ and $w_\text{s}$. Empty markers depict the APR, i.e. the TPR using solely the analytical formulae of the witnesses.

Subsequently, we have obtained the confusion matrices for all these SVCs based on their decision on the test set (independent on the training set). TPR and FPR are then directly calculated from these matrices and plotted in Fig. 3. By splitting the test set into 50 batches and evaluating them independently, we were able to estimate standard deviation of all the presented quantities. As a result we obtain complete ROC curves for the idea of purity-enhanced classifications. Areas under curve (AUC) for all three witnesses are numerically calculated to evaluate the performance of this method (see Table 1). We have found out, that using this approach, one can improve the TPR typically by a factor of 1.3 by only sacrificing less than 1‰ of specificity (FPR < 0.001). Experimental imperfections and detection noise will inevitably cause even theoretically perfect witnesses to become imperfect. Considering that we believe that the price of a quite small specificity drop is worth the tangibly increased sensitivity. Moreover, we have demonstrated that sensitivity can further be improved to near TPR close to 1 at the expense of decreased specificity. This is well visible in the presented ROC curves whose AUC is, for all tested witnesses, greater than 0.9. For all numerical results see Supplementary Tables 2–4 in the Supplementary Material. The amount of specificity that one is willing to sacrifice to boost the TPR is strongly application-dependent. While there does not exist any general optimal point, it is reasonable to assume that decreased specificity can be tolerated at least to such extent where it results in comparable amount of misclassifications as induced by other imperfections of the specific application (such as detection noise due to finite acquisition time or imprecision in the measurement setting). Improving TPR at the expense of specificity beyond this point is to be considered by the users based on the their particular goals. One can for instance significantly boost TPR in order to intelligently pre-select potentially entangled states that are subsequently tested by another procedure.

**Table 1:** Values of areas under the curves (AUC) and analytical positive rates (APR) for tested witnesses.

	AUC	APR (%)
Collectibility	$0.902 \pm 0.002$	$16.7 \pm 0.4$
CHSH witness	$0.965 \pm 0.001$	$45.5 \pm 0.5$
Entropic witness	$0.973 \pm 0.001$	$58.8 \pm 0.6$

**Figure 4:**
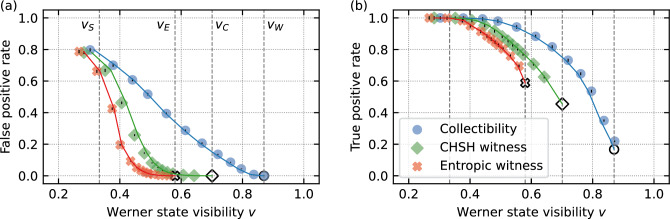
Classification of experimental Werner states $\rho _W(v)$ by means of the SVCs based on the Collectibility, CHSH and Entropic witnesses complemented by purity. The plot visualizes the minimal visibilities $v_\text{ex}$ of Werner states classified as entangled and the corresponding (**a**) FPR and (**b**) TPR of the classifier. Solid lines represent corresponding minimal visibilities $v_\text{th}$ for perfect Werner states (ideal correlation matrices *R*). Empty markers and the vertical dashed lines denote the minimum visibilities of detected entangled Werner states by the respective analytical witnesses. The ideal Werner states are separable for $v_S\le 1/3$.

### Results on experimental Werner states

In the next step, we evaluate the performance of the SVCs on real experimental data. For this purpose, we consider the scenario already described in Section Vulnerability of collective measurements to white noise, namely, we deal with two copies of the Bell state $\hat{\text {P}}^+$ affected by the depolarising channel. As a result, each output state is described by $\rho _W(v) = v~\hat{\text {P}}^+ + (1-v) \hat{\mathbbm {1}}/4$, where the parameter $1-v$ quantifies a noise level (c.f. Eq. (2)). We test the SVC procedure on a set of the experimental Werner states $\rho _W$ with increasing amounts of noise (decreasing *v*). It shall be stressed out that we deploy the SVC models trained solely on numerical data as explained in the previous subsection. We have not resorted to any retraining of the models using experimental data, hence the training of the models does not consume any experimental resources at all. As we show in this subsection, models trained purely on numerical states perform quite well when classifying real-experiment measurement outcomes. Werner states were generated and subjected to the collective measurements in our previously published experimental setup^46^. The experiment was implemented on the platform of linear optics using polarization degree of freedom of two pairs of photons (for more details on the experimental setup and measurement procedure see Section III and IV in Ref.^46^). The performed polarization projections and counting of four-fold detection events allowed us to acquire the values of $C (\hat{\Pi }_{\text {A}_{\text {1}}},\hat{\Pi }_{\text {A}_{\text {2}}})$ and $\bar{C} (\hat{\Pi }_{\text {A}_{\text {1}}},\hat{\Pi }_{\text {A}_{\text {2}}})$ for Werner states of given visibilities and subsequently to calculate the values of all the three witnesses presented in this paper. The state purity is determined from the collective measurements as well.

The inclusion of estimated purity in the SVC enables decreasing the threshold value for a minimum visibility that leads to correct classification of entangled Werner states. Our results are presented in Fig. 4, where the empty markers depict minimum visibility for the analytical witnesses and the filled markers and lines depict minimum visibility of experimental ($v_\text{ex}$) and ideal ($v_\text{th}$) Werner states classified as entangled as functions of the FPR and TPR of the SVC. There exists a trade-off between the minimum visibility of correctly classified entangled Werner state and the specificity of the classifier expressed in terms of the FPR. For example, the SVC using the CHSH witness and purity classifies correctly Werner states with $v\ge 0.61$ at the expense of only FPR=0.39 %. Note that the analytical CHSH witness has the detection threshold at $v_C=\frac{1}{\sqrt{2}}\approx 0.71$. For all numerical results see Supplementary Tables 2–4 in the Supplementary Material.

Analysis of the results depicted in the Fig. 4 clearly indicates that one can adjust the penalties $w_e$ and $w_s$ so that Werner states of visibility $v\ge v_{\text{th}}$ are positively identified as entangled ($v_{\text{th}}$ being an arbitrary detection visibility threshold). It may be tempting to set $v_{\text{th}} = v_S = 1/3$ so that all entangled Werner states are detected and no separable entangled state get misclassified. Such approach would, however, result in a rather large FPR for all other states (see values of FPR in Fig. 4a for visibilities *v* approaching $v_S$).

This example clearly demonstrates the suitability of our method for detecting entangled states even in the presence of relatively strong noise. Let us note, however, that the entanglement detection method described above cannot be freely extended to other types of quantum correlations, e.g. Bell’s nonlocality. In this case, $v_C$ cannot be exceeded as such a result has no physical meaning. Nonetheless, our findings open the door for further studies in this direction.

## Discussion

We have demonstrated that white noise significantly impairs the performance of collective entanglement witnesses. This effect was documented on the example of Collectibility loosing the capability to detect entangled Werner states when noise level exceeds 13 %. To mitigate this shortcoming, we have proposed to correlate the values of the collective witnesses with the state purity. Achieving this task analytically proves to be impractical, especially considering the need to tune detection specificity. The solution lies in employing machine learning.

The theoretical result on the sensitivity of collective measurement to white noise that is mathematically expressed in the form of Eq. (3) and the distribution of entangled states in Fig. 2 are in mutual agreement. Considering that insertion of white noise typically reduces purity, the entangled states have a tendency to distribute along the diagonal in the Fig. 2 documenting a corelation between purity (insertion of white noise) and the values of the three collective witnesses. This effect is perhaps the best visible in the case of the CHSH witness (Fig. 2b) or in the Supplementary Figs. 5–7 in the Supplementary Material. It becomes evident that the corelation between purity and the values of collective witnesses can be explored to boost the TPR of entanglement detection. The specific shape of the dispersion of entangled states in the space spanned by purity and the collective witness is, however, nontrivial and warrants application of machine learning to find optimal solutions, i.e. maximum sensitivity for a given detection specificity. Reaching perfect specificity is no longer possible, because at some point the entangled states with reduced purity enter the region where separable states already start appearing. We, thus, resort to parametrization of the increased sensitivity either by the TPR or the improvement factor (IF), i.e. the improvement factor over classification based solely on the collective witnesses without correlating with purity. Similarly we parametrize the decreased specificity by the FPR and finally reconstruct the ROC curves of the entire procedure.

Specifically we have implemented SVC models to optimize the decision boundary between entangled and separable states in the feature space spanned by the values of the entanglement witness and the states’ purities. Our findings indicate that the range of detected entangled states can be expanded by a significant percentage at a minimal cost in specificity. To further promote the strength of our method, we have included classification of experimental Werner states. We have demonstrated that a considerable increase in the range of correctly classified Werner states can be accomplished at a relatively small cost in specificity of the classifiers. Consequently, we believe that the idea of correlating entanglement witnesses with purity holds promise for practical near-future quantum communications, particularly in the context of entanglement swapping as showcased in this paper. It can be interesting to investigate the decision-making using SVC trained in spaces of other quantum state quantifiers besides purity and the selected collective witnesses. Perhaps additional improvement can be reached when multiple witnesses are used as coordinates of the decision space. Similarly interesting might be the transition to a higher dimension or to a multipartite system^47–49^, where entanglement detection becomes significantly more challenging. The question of whether the presented method can benefit other technologies beyond quantum communications is also pertinent. Although these are definitely inspiring propositions, they are already out of the scope of this paper.

### Supplementary Information

Supplementary Information.

## Data Availability

The Supplementary Material referenced in the text is available at the publisher’s website. The datasets and source codes generated and/or analysed during the current study are available in the Figshare repository, http://dx.doi.org/10.6084/m9.figshare.25604676
