# Supplementary material for: Enhancing collective entanglement witnesses through correlation with state purity
Source: Sci Rep. 2024 Jul 16;14:16374. doi: 10.1038/s41598-024-65385-7 (PMC11252302; doi:10.1038/s41598-024-65385-7)
Supplement: Supplementary file 1 — Supplementary Information. [file 41598_2024_65385_MOESM1_ESM.pdf]

Enhancing Collective Entanglement Witnesses through Correlation with State Purity

Kateřina Jiráková[1], Antonín Černoch[1], Artur Barasiński[2], and Karel Lemr[3,*]

[1]Institute of Physics of the Academy of Sciences of the Czech Republic, Joint Laboratory of Optics of Palacký University and Institute of Physics AS CR, 17. listopadu 50a, 772 07 Olomouc, Czech Republic
[2]Institute of Theoretical Physics, University of Wroclaw, Plac Maxa Borna 9, 50-204 Wroclaw, Poland
[3]Palacký University in Olomouc, Faculty of Science, Joint Laboratory of Optics of Palacký University and Institute of Physics AS CR, 17. listopadu 12, 771 46 Olomouc, Czech Republic
[*]k.lemr@upol.cz

Supplementary Material

Preparing data sets

There are several methods to generate random density matrices. These methods usually employ some density matrix distance measure (Hilbert-Schmidt or Bures) and try to obtain uniform distribution of these distances over generated states, see Supplementary Figure 1. Another method involves random diagonal matrices subjected to global unitary rotations[1,2]. There are dedicated libraries in Python implementing random state generation, for example
`qiskit.quantum_info.random_density_matrix()`[3] or `qutip.rand_dm()`[4].

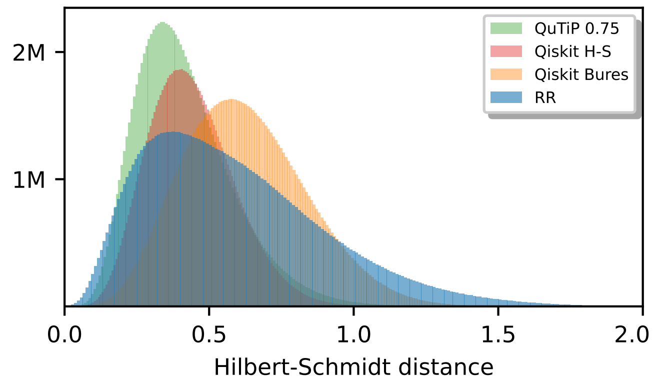

Supplementary Figure 1. Histogram of Hilbert-Schmidt distances (squared) of all combinations between 10.000 random states generated by QuTiP library with parameter `density=0.75`, Qiskit library with two different settings of parameter `method='Hilbert-Schmidt','Bures'` and by random rotation (RR) method (*200 bins*).

These methods, in general, do not deliver states such as pure separable or pure entangled with sufficient prevalence because of their uncommon presence in the Hilbert space. The absence of pure states would overestimate the power of our method because we compare it with the analytical function of the witnesses that perform well on pure states. To deal with this effect and make the conditions more challenging we decided to prepare a training data set with equally distributed purity where half of the states are entangled (negativity $N > 0$) and the second not ($N = 0$). This way we make sure that our method is fairly evaluated with respect to whatever conditions a user might have.

For generation of states uniformly distributed in purity we used the method of random global rotations because of its broad distribution in Hilbert-Schmidt distances. During states generation we control the values of purity (in 0.01 binning in interval [0.25,1]) and negativity (binary – $N = 0$ or $N \neq 0$) and discard states which occur too frequently. Finally we obtain two million density matrices for which the values of negativity, purity, Collectibility, CHSH and Entropic witnesses were calculated. The histograms of these values are presented in Supplementary Figures 2, 3 and 4.

Support vector machine (SVM) tries to find the border hyperplane between entangled and separable states knowing the values of purity and the analytical value of an entanglement witness. To assess the SVM decision we can visualize density maps (in logarithmic scale) of purity and witnesses under the consideration for separable and entangled states separately, see Supplementary Figures 5, 6 and 7.

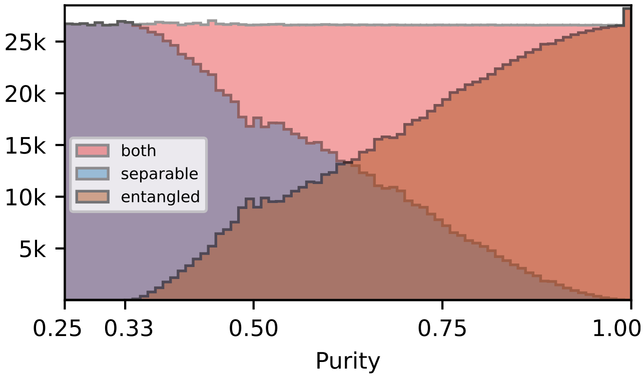

Supplementary Figure 2. Purity histogram of training and testing dataset consisting of 2 millions of states (*75 bins*).

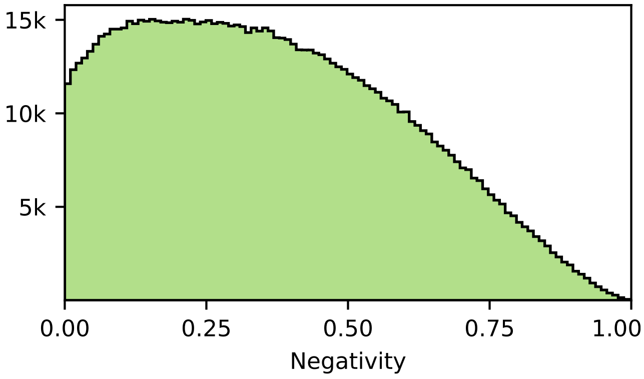

Supplementary Figure 3. Negativity histogram of training and testing dataset consisting of 1 million entangled states (*100 bins*).

TPR and FPR values

The decision of SVM results in a confusion matrix. On its diagonal lie true recognitions of entangled (true positive - TP) and separable (true negative - TN) states. Off-diagonal terms represent wrong assignment, false negative (FN) when entangled states were marked as separable and false positive (FP) whereas separable states were marked as entangled. True positive rate (TPR) and false positive rate (FPR) forming receiver operating characteristic (ROC) curve are calculated directly from the confusion matrix:

$$\begin{pmatrix} \mathrm{TP} & \mathrm{FN} \\ \mathrm{FP} & \mathrm{TN} \end{pmatrix}, \quad \mathrm{TPR} = \frac{\mathrm{TP}}{\mathrm{TP}+\mathrm{FN}}, \quad \mathrm{FPR} = \frac{\mathrm{FP}}{\mathrm{FP}+\mathrm{TN}}.$$

Upon training of the SVM the class-specific penalties w_e and w_s are tuned accordingly to homogeneously cover the entire interval of the ROC. The specific values of these penalties are presented in Supplementary Table 1. For each point we can also calculate the improvement factor (IF) which quantifies how much bigger portion of entangled states is recognized by the SVM when the value of purity is included in the decision. Naturally, this improvement is accompanied by the increments of misclassified separable states.

Results are summarized in Tabs. 2 – IV.

Supplementary Table 1. Pairs of penalties w_e and w_s used for classification.

w_e	5.62	2.82	1.78	1.41	1.19	1.00	0.84	0.67	0.50	0.35	0.20	0.07
w_s	0.18	0.35	0.56	0.71	0.84	1.00	1.19	1.50	2.00	2.82	5.01	14.13

Supplementary Table 2. Confusion matrices, values of true positive rates (TPR) and false positive rates (FPR) for different improvement factors (IF) of SVCs trained on the values of purity and the Collectibility. Minimum visibility of experimental v_{ex} and ideal v_{th} Werner states classified as entangled are included in the last two columns.

IF	confusion matrix	TPR (%)	FPR (%)	v_{th}	v_{ex}
1.31±0.04	$\begin{pmatrix} 109389 & 390616 \\ 342 & 499653 \end{pmatrix}$	21.9±0.5	0.07±0.02	0.86	0.87
2.10±0.06	$\begin{pmatrix} 174947 & 325058 \\ 4120 & 495875 \end{pmatrix}$	35.0±0.6	0.82±0.08	0.83	0.84
3.21±0.09	$\begin{pmatrix} 267851 & 232154 \\ 21849 & 478146 \end{pmatrix}$	53.6±0.9	4.7±0.2	0.79	0.80
3.9±0.1	$\begin{pmatrix} 323142 & 176863 \\ 42900 & 457095 \end{pmatrix}$	64.6±0.9	8.6±0.3	0.75	0.76
4.4±0.1	$\begin{pmatrix} 365933 & 134072 \\ 67859 & 432136 \end{pmatrix}$	73.2±0.9	13.6±0.3	0.71	0.72
4.9±0.1	$\begin{pmatrix} 408305 & 91700 \\ 103367 & 396628 \end{pmatrix}$	82.0±1.0	20.7±0.4	0.66	0.67
5.3±0.1	$\begin{pmatrix} 441720 & 58285 \\ 143770 & 356225 \end{pmatrix}$	88±1	28.8±0.5	0.61	0.61
5.7±0.1	$\begin{pmatrix} 471245 & 28760 \\ 197114 & 302881 \end{pmatrix}$	94±1	39.4±0.6	0.55	0.55
5.9±0.1	$\begin{pmatrix} 489877 & 10128 \\ 258399 & 241596 \end{pmatrix}$	98±1	51.7±0.8	0.49	0.49
6.0±0.1	$\begin{pmatrix} 496859 & 3146 \\ 303811 & 196184 \end{pmatrix}$	99±1	60.8±0.9	0.44	0.44
6.0±0.2	$\begin{pmatrix} 499555 & 450 \\ 350121 & 149874 \end{pmatrix}$	100±1	70.0±1.0	0.39	0.38
6.0±0.1	$\begin{pmatrix} 500005 & 0 \\ 398691 & 101304 \end{pmatrix}$	100±1	80±1	0.32	0.30

Supplementary Table 3. Confusion matrices, values of true positive rates (TPR) and false positive rates (FPR) for different improvement factors (IF) of SVCs trained on the values of purity and the CHSH witness. Minimum visibility of experimental v_{ex} and ideal v_{th} Werner states classified as entangled are included in the last two columns.

IF	confusion matrix	TPR (%)	FPR (%)	v_{th}	v_{ex}
1.38±0.02	$\begin{pmatrix} 313017 & 186988 \\ 302 & 499693 \end{pmatrix}$	62.6±0.8	0.06±0.02	0.64	0.64
1.56±0.03	$\begin{pmatrix} 353662 & 146343 \\ 1925 & 498070 \end{pmatrix}$	70.7±0.9	0.39±0.06	0.61	0.61
1.69±0.03	$\begin{pmatrix} 384928 & 115077 \\ 6168 & 493827 \end{pmatrix}$	77.0±0.9	1.2±0.1	0.58	0.57
1.77±0.03	$\begin{pmatrix} 401385 & 98620 \\ 10774 & 489221 \end{pmatrix}$	80.3±0.9	2.2±0.2	0.56	0.56
1.82±0.03	$\begin{pmatrix} 414159 & 85846 \\ 16624 & 483371 \end{pmatrix}$	83.0±1.0	3.3±0.2	0.54	0.54
1.88±0.03	$\begin{pmatrix} 427452 & 72553 \\ 25851 & 474144 \end{pmatrix}$	85.0±1.0	5.2±0.2	0.53	0.52
1.94±0.03	$\begin{pmatrix} 441112 & 58893 \\ 40721 & 459274 \end{pmatrix}$	88.0±1.0	8.1±0.3	0.51	0.51
2.02±0.03	$\begin{pmatrix} 458795 & 41210 \\ 71985 & 428010 \end{pmatrix}$	92±1	14.4±0.4	0.49	0.48
2.10±0.03	$\begin{pmatrix} 476498 & 23507 \\ 128849 & 371146 \end{pmatrix}$	95±1	25.8±0.6	0.46	0.45
2.16±0.03	$\begin{pmatrix} 491819 & 8186 \\ 231088 & 268907 \end{pmatrix}$	98±1	46.2±0.8	0.42	0.41
2.20±0.03	$\begin{pmatrix} 499293 & 712 \\ 333931 & 166064 \end{pmatrix}$	100±1	66.8±1.0	0.37	0.35
2.20±0.03	$\begin{pmatrix} 499978 & 27 \\ 390472 & 109523 \end{pmatrix}$	100±1	78±1	0.31	0.28

Supplementary Table 4. Confusion matrices, values of true positive rates (TPR) and false positive rates (FPR) for different improvement factors (IF) of SVCs trained on the values of purity and the Entropic witness. Minimum visibility of experimental v_{ex} and ideal v_{th} Werner states classified as entangled are included in the last two columns.

IF	confusion matrix	TPR (%)	FPR (%)	v_{th}	v_{ex}
1.18±0.02	$\begin{pmatrix} 347708 & 152297 \\ 168 & 499827 \end{pmatrix}$	69.5±0.9	0.03±0.02	0.56	0.56
1.29±0.02	$\begin{pmatrix} 379263 & 120742 \\ 1227 & 498768 \end{pmatrix}$	75.9±0.9	0.25±0.05	0.53	0.53
1.37±0.02	$\begin{pmatrix} 401933 & 98072 \\ 4285 & 495710 \end{pmatrix}$	80.4±0.9	0.9±0.1	0.51	0.51
1.41±0.02	$\begin{pmatrix} 413866 & 86139 \\ 7479 & 492516 \end{pmatrix}$	82.8±0.9	1.5±0.1	0.49	0.49
1.44±0.02	$\begin{pmatrix} 423261 & 76744 \\ 11424 & 488571 \end{pmatrix}$	84.7±0.9	2.3±0.2	0.48	0.48
1.47±0.02	$\begin{pmatrix} 432815 & 67190 \\ 17265 & 482730 \end{pmatrix}$	86±1	3.5±0.2	0.47	0.47
1.51±0.02	$\begin{pmatrix} 442813 & 57192 \\ 26179 & 473816 \end{pmatrix}$	88.6±1.0	5.2±0.2	0.46	0.46
1.55±0.02	$\begin{pmatrix} 457069 & 42936 \\ 46544 & 453451 \end{pmatrix}$	91±1	9.3±0.3	0.44	0.44
1.61±0.02	$\begin{pmatrix} 474819 & 25186 \\ 97820 & 402175 \end{pmatrix}$	95±1	19.6±0.5	0.41	0.40
1.67±0.02	$\begin{pmatrix} 491159 & 8146 \\ 212344 & 287651 \end{pmatrix}$	98±1	42.5±0.8	0.38	0.38
1.70±0.02	$\begin{pmatrix} 499314 & 691 \\ 330981 & 169014 \end{pmatrix}$	100±1	66.2±1.0	0.34	0.33
1.70±0.02	$\begin{pmatrix} 499994 & 11 \\ 393410 & 106585 \end{pmatrix}$	100±1	79±1	0.28	0.27

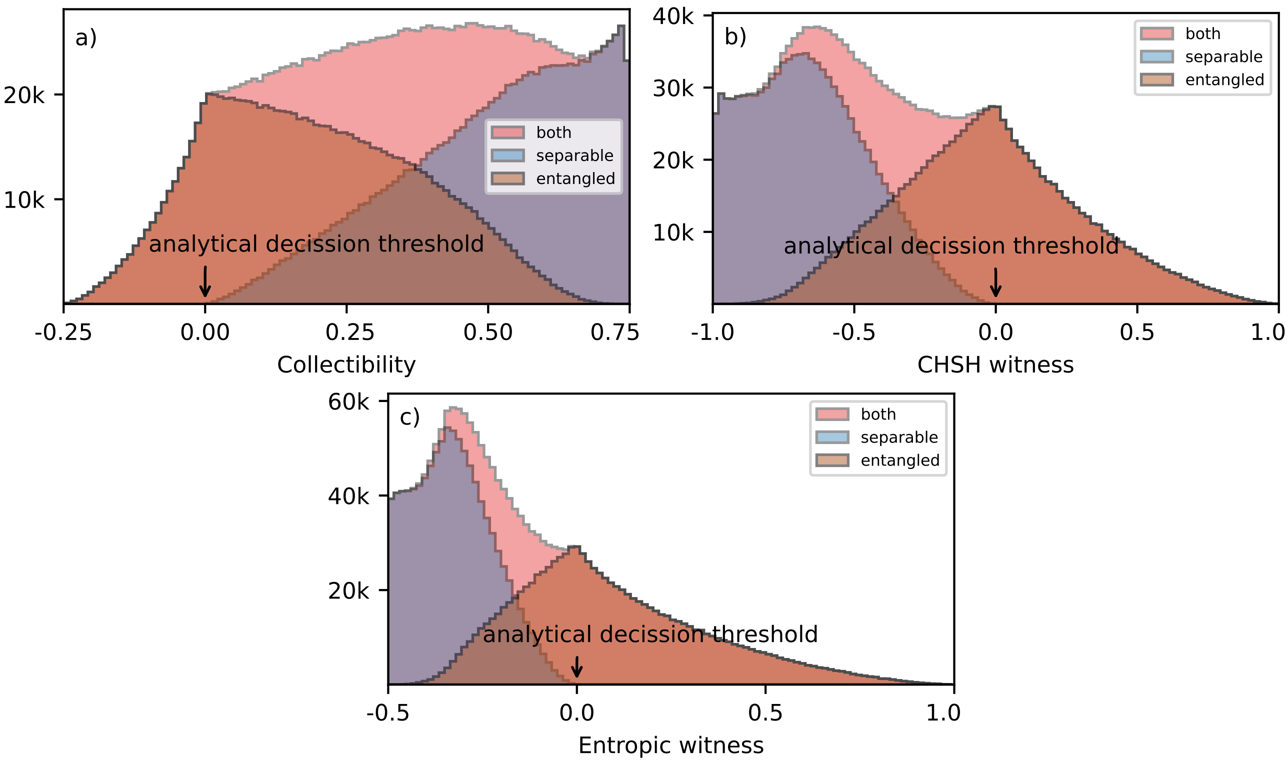

Supplementary Figure 4. Histograms of entanglement witnesses applied on training and testing dataset consisting of 2 millions of states, a) Collectibility, b) CHSH and c) Entropic witnesses (*100 bins*).

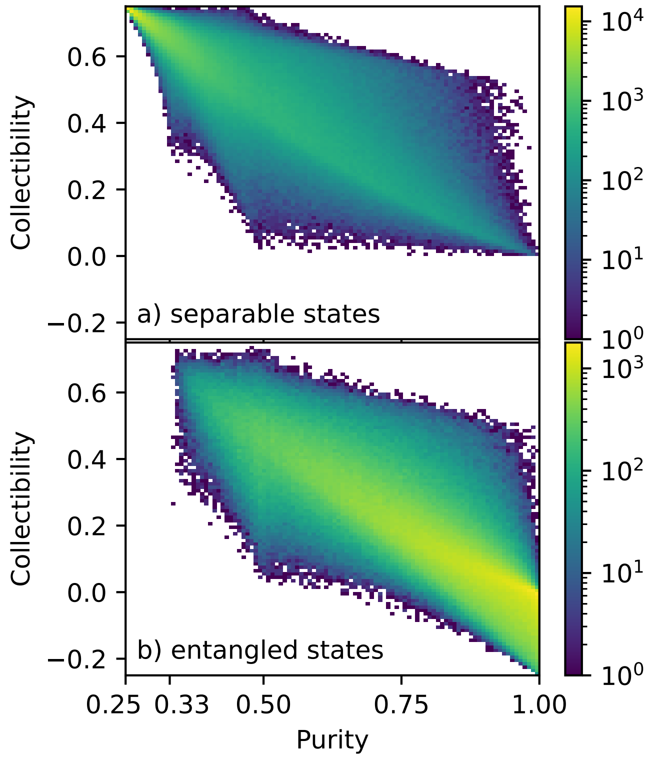

Supplementary Figure 5. Density maps of purity vs Collectibility of training and testing dataset consisting of a) 1 million separable and b) 1 million entangled states.

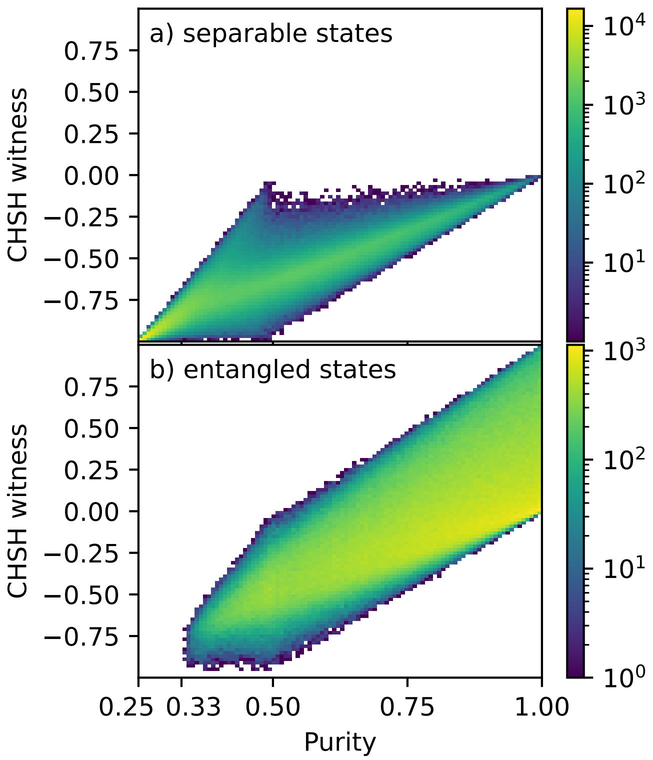

Supplementary Figure 6. Density maps of purity vs CHSH witness of training and testing dataset consisting of a) 1 million separable and b) 1 million entangled states.

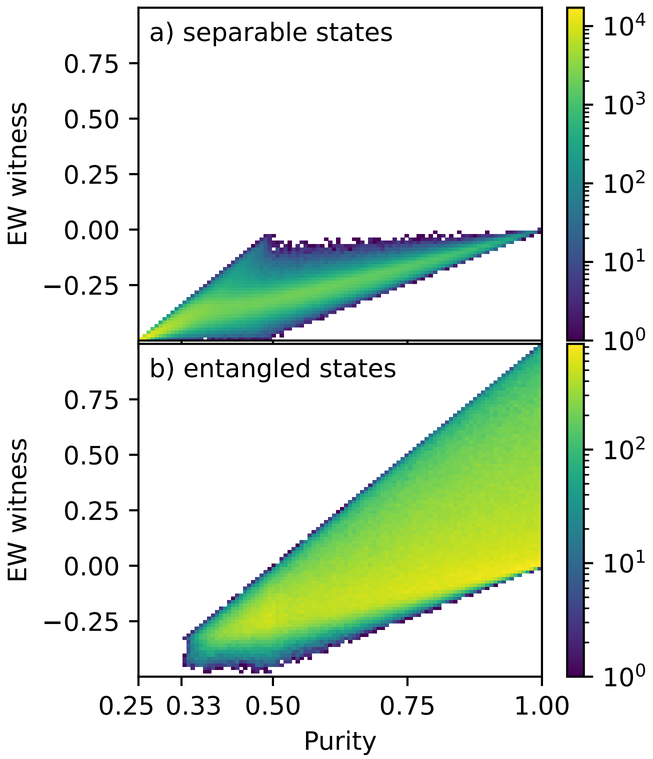

Supplementary Figure 7. Density maps of purity vs Entropic witness of training and testing dataset consisting of a) 1 million separable and b) 1 million entangled states.

References

1. Maziero, J. Random sampling of quantum states: a survey of methods. *Braz. J. Phys.* **45**, 575–583, DOI: 10.1007/s13538-015-0367-2 (2015).
2. Li, C.-K., Roberts, R. & Yin, X. Decomposition of unitary matrices and quantum gates. *Int. J. Quantum Inf.* **11**, 1350015, DOI: 10.1142/S0219749913500159 (2013).
3. Qiskit contributors. Qiskit: An open-source framework for quantum computing, DOI: 10.5281/zenodo.2573505 (2023).
4. Johansson, J., Nation, P. & Nori, F. Qutip 2: A Python framework for the dynamics of open quantum systems. *Comput. Phys. Commun.* **184**, 1234–1240, DOI: https://doi.org/10.1016/j.cpc.2012.11.019 (2013).
